# Genetic characterization and phylogenetic analysis of the *Nigella sativa* (black seed) plastome

**DOI:** 10.1038/s41598-024-65073-6

**Published:** 2024-06-24

**Authors:** Lubna, Sajjad Asaf, Ibrahim Khan, Rahmatullah Jan, Saleem Asif, Saqib Bilal, Kyung-Min Kim, Ahmed AL-Harrasi

**Affiliations:** 1 Natural and Medical Science Research Center, University of Nizwa, 616 Nizwa, Oman; 2 Department of Applied Biosciences, Kyungpook National University, Daegu, 41566 Republic of Korea

**Keywords:** *N. sativa*, Plastome, Divergence, Hotspot regions, Phylogenetic study, Plant sciences, Plant genetics

## Abstract

In this study, the complete plastome sequence of *Nigella sativa* (black seed), was analyzed for the first time. The plastome spans approximately 154,120 bp, comprising four sections: the Large Single-Copy (LSC) (85,538 bp), the Small Single-Copy (SSC) (17,984 bp), and two Inverted Repeat (IR) regions (25,299 bp). A comparative study of *N. sativa*’s plastome with ten other species from various genera in the Ranunculaceae family reveals substantial structural variations. The contraction of the inverted repeat region in *N. sativa* influences the boundaries of single-copy regions, resulting in a shorter plastome size than other species. When comparing the plastome of *N. sativa* with those of its related species, significant divergence is observed, particularly except for *N. damascena*. Among these, the plastome of *A. glaucifolium* displays the highest average pairwise sequence divergence (0.2851) with *N. sativa*, followed by *A. raddeana* (0.2290) and *A. coerulea* (0.1222). Furthermore, the study identified 12 distinct hotspot regions characterized by elevated Pi values (> 0.1). These regions include *trn*H*-GUG-psb*A*, mat*K*-trn*Q*-UUG, psb*K*-trn*R*-UCU, atp*F*-atp*I*, rpo*B*-psb*D*, ycf3-ndh*J*, ndh*C*-cem*A*, pet*A*-psa*J*, trn*N-*GUU-ndh*F*, trn*V*-GAC-rps12, ycf2-trn*I*-CAU, and ndh*A*-ycf1*. Approximately, 24 tandem and 48 palindromic and forward repeats were detected in *N. sativa* plastome. The analysis revealed 32 microsatellites with the majority being mononucleotide repeats. In the *N. sativa* plastome, phenylalanine had the highest number of codons (1982 codons), while alanine was the least common amino acid with 260 codons. A phylogenetic tree, constructed using protein-coding genes, revealed a distinct monophyletic clade comprising *N. sativa* and *N. damascene*, closely aligned with the Cimicifugeae tribe and exhibiting robust support. This plastome provides valuable genetic information for precise species identification, phylogenetic resolution, and evolutionary studies of *N. sativa*.

## Introduction

Chloroplast genome (plastome) comparative analysis has proven to be a valuable tool in phylogeny reconstruction and resolving complex evolutionary relationships^1–5^. In angiosperms, it has been observed that the number and order of genes in the plastome are generally conserved^6^. This conservation is attributed to the relatively slower evolution rate of chloroplast sequences compared to nuclear regions^7,8^. However, it is worth noting that sequence rearrangements in plastome have been reported in various plant species^9–11^. Inverted repeats region (IR) expansions or contractions into single-copy areas containing inversions, as well as significant inversions in large single-copy regions (LSC), are some examples of these rearrangements^12,13^. These inversion occurrences were most likely caused by intragenomic recombination in areas with varying G + C concentrations^14,15^ or tRNA activity^16^. The importance of gene rearrangements and inversions in plastomes for phylogenetic analyses lies in their rarity, ease of homology estimation, and simplicity in determining the polarity of inversion events^17–19^. The comparisons facilitate the investigation of molecular evolutionary patterns linked to structural rearrangement and the clarification of the molecular mechanisms responsible for those occurrences.

With a global distribution, the Ranunculaceae family has about 2000 primarily herbaceous species^20–22^ and is considered one of the oldest families to diverge from the eudicots. It is a large family, which includes approximately 59 genera and numerous Ranunculaceae plants have significant medicinal uses^23^. Deep discoveries and a reevaluation of the taxonomy of Ranunculaceae have been made possible in recent years by molecular phylogenetics. The results of molecular phylogenetic research have led to the reduction of several genera and the proposal of a new genus^21,24–28^. Several widely used plastid regions and tandemly repeated DNA have been the primary data sources for all molecular research conducted to date plastomes. Few entire plastomes have been published and made available through GenBank (http://www.ncbi.nlm.nih.gov).

*Nigella*, commonly known as fennel flower, constitutes a compact genus within the Nigelleae tribe, comprising 18 species in the Ranunculaceae family^29,30^. This genus is indigenous to Southern Europe, North Africa, South Asia, Southwest Asia, and the Middle East^31,32^. *Nigella* comprises fourteen species, *N. sativa *L. (black cumin) stands out as the most popular medicinal plant. Moreover, the seeds of *N. sativa *L. are utilized as spices in various culinary applications. *N. damascena *L. and *N. arvensis* are annual plants known for their ornamental and medicinal qualities^33–35^. A limited number of studies have examined genetic variation in *N. sativa* (black cumin) using DNA-based molecular markers^36,37^. Plastid phylogenomic investigations can be especially effective in elucidating the generic relationships within the Ranunculaceae family. Structural variations in the plastome, such as gene inversions, gene transpositions, and expansion–contraction of the inverted repeat (IR), offer valuable systematic insights into the family^22,38,39^.

In this study, we sequenced, assembled, and analyzed the complete plastome sequence of the *N. sativa* plant for the first time, which belongs to the Ranunculaceae family. We compared it with ten previously published chloroplast genome sequences from the Ranunculaceae family obtained from the National Center for Biotechnology Information (NCBI). This study conducted a general characteristic analysis of plastome for all species and compared it with *N. sativa*. This analysis likely encompassed a thorough examination of various features such as structure, gene composition, and other relevant attributes within the plastome of the studied species. Furthermore, the study involved the identification of microsatellites (SSRs), long repeat sequences, and highly variable regions within the chloroplast genomes of *N. sativa* and other studied species.

## Results

### General features and composition of plastome

**Figure 1:**
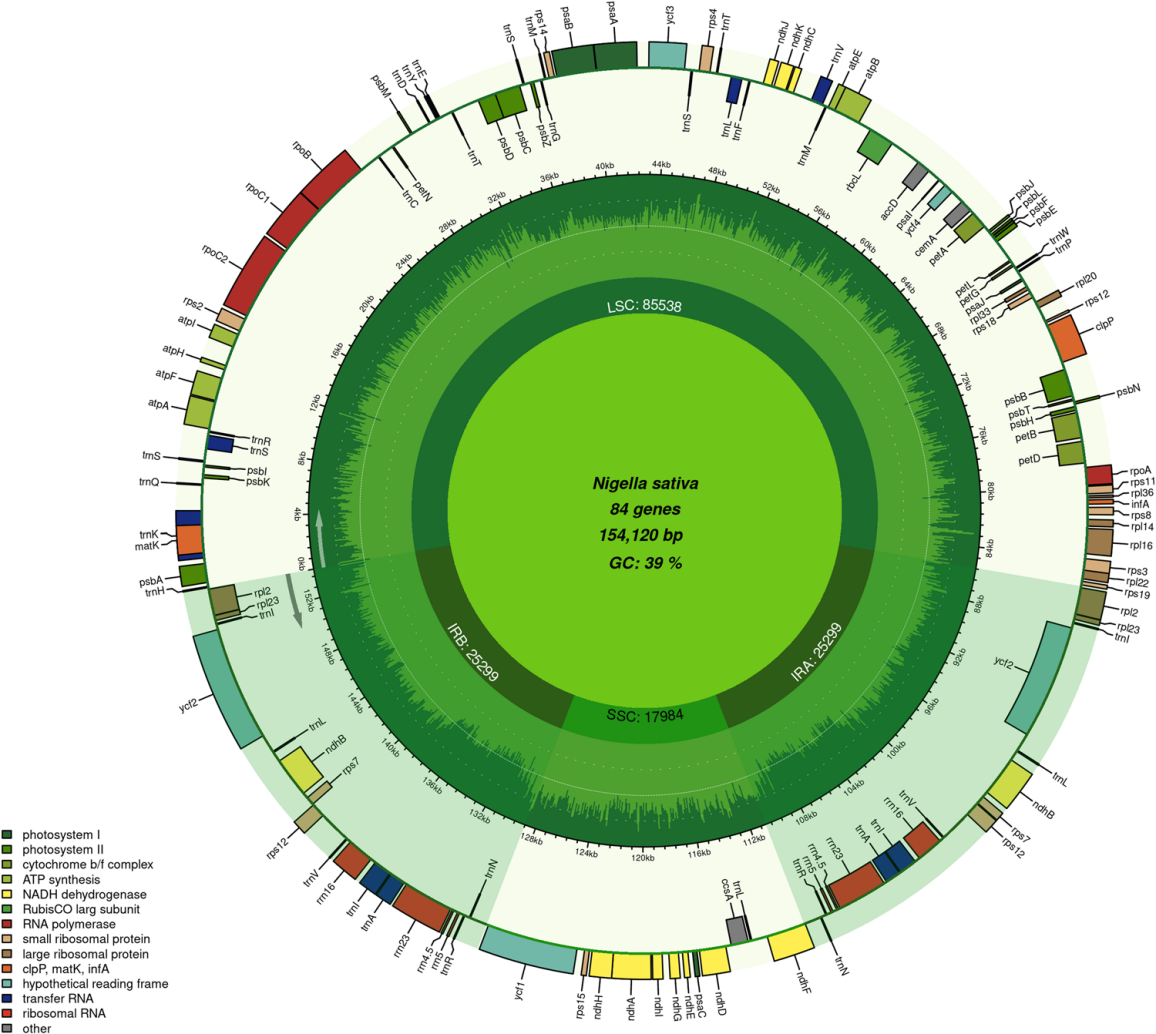
Plastome genome map of *N. sativa*. Genes drawn outside the circle are transcribed anti-clockwise, while those inside the circle are transcribed clockwise. Large single copy (LSC) region, inverted repeat (IRA, IRB) regions and small single copy (SSC) region are shown in the figure. The darker green color in the inner circle corresponds to GC content whereas the lighter green corresponds to AT content. Different colors of genes represent their different functions.

This research investigates the plastome structure of *N. sativa* and compares it with the plastomes of ten additional species within the Ranunculaceae family. The complete plastome of *N. sativa* exhibits a quadripartite structure, consistent with the typical organization found in most land plant plastomes (Fig. 1). The plastome of *N. sativa* is approximately 154,120 bp in size and is divided into four main sections. These include the LSC region, which spans 85,538 bp, the SSC region covering 17,984 bp, and two IR regions with a total size of 25,299 bp. In this study, the plastome of *P. anemonoides* emerged as the largest, spanning a length of 164,383 bp, whereas the plastome of *N. sativa* was identified as the shortest among the 11 selected plastomes. The plastome of *N. sativa* contains a total of 128 genes, consisting of 83 genes for encoding proteins, 37 genes for transfer RNA (tRNA), and eight genes for ribosomal RNA (Table 1). The gene count for this organism is the most minimal among all plastomes, with *A. coerulea* displaying a larger total of 140 genes. There is variability in the number of protein-coding genes across the studied species, ranging from 81 to 94. Notably, *N. sativa* possesses a total of 83 protein-coding genes. Upon examining all species in the study, it is evident that *A. glaucifolium* boasts the highest number of protein-coding genes (PCGs), while *A. coerulea* exhibits the lowest count of PCGs. Within the plastome of *N. sativa*, 11 genes (*rps*11*, rps*12*, rps*14*, rps*15*, rps*18*, rps*19*, rps*2*, rps*3*, rps*4*, rps*7 and *rps*8) encode for small ribosomal subunits, while another set of eight genes (*rpl*14*, rpl*16*, rpl*2*, rpl*20*, rpl*22*, rpl*23*, rpl*33 and *rpl*36) encode for large ribosomal subunits. Furthermore, there are 45 genes associated with proteins related to photosynthesis, and an additional four genes (*rpo*A, *rpo*B, *rpo*C1, and *rpo*C2) are involved in encoding DNA-dependent RNA polymerase. Lastly, nine genes (*acc*D, *ccs*A, *cem*A, *mat*K, *clp*P, *inf*A, *ycf*1, *ycf*2, and *ycf*4) are associated with the encoding of other proteins, as outlined in Table 2. The tRNA gene count ranges from 36 (in *A. glaucifolium* and *A. raddeana*) to 45 (in *A. coerulea*), while the rRNA gene count remains constant at 8 across all plastomes. We found 11 intron-containing genes (*atp*F*, ndh*A*, ndh*B*, pet*B*, pet*D*, rpl*16*, rpl*2, and *rpo*C1) in *N. sativa* plastome, eight of which contained single intron, whereas three genes (*clp*P, *rps*12 and *ycf*3) have two introns each (Table 3). The GC content of the plastome among the 11 species was generally similar, with *N. sativa* exhibiting a GC percentage of approximately 38%. In contrast, *A. coerulea* displayed a higher GC content of 39% across all the plastomes examined. In examining PCG length in *N. sativa* plastome, we found a length of 76,339 bp. Comparative analysis across species revealed diverse PCG lengths, ranging from 75,870 bp (*N. damascene*) to 84,105 bp (*A. glaucifolium*). Additionally, IR lengths in plastomes varied from 31,279 bp (*A. raddeana*) to 25,162 bp (*N. damascene*), indicating a positive correlation between overall plastome length and IR size across species (Table 1). We examined the codon usage frequency of protein-coding genes in the *N. sativa* plastome; phenylalanine had the most codons (1982 codons), then Lysine (1912 codons), while Alanine was the least common amino acid (260 codons). Of the total codons analyzed, 35 exhibited a relative synonymous codon usage (RSCU) greater than 1 in the *N. sativa* plastome. The most favored codon was AGA, encoding arginine, with an RSCU value of 1.78. Following closely, CAU, which encodes histidine, had an RSCU value of 1.44 (Table S1).

**Table 1:** Basic features of the plastome of the *N. sativa* species and related species.

Name	Genome size	%GC	LSC Size	SSC size	IR size	Number of total genes	Protein coding genes	tRNA genes	rRNA genes	PCD size	Genes with introns
*N. sativa*	154,120	38.80	85,538	17,984	25,299	128	83	37	8	76,339	15 + 8
*N. damascena*	155,218	38.80	87,113	17,783	25,162	129	83	37	8	75,870	15 + 8
*A. angustius*	156,109	38.00	86,719	16,940	26,225	128	82	38	8	77,400	14 + 8
*A. asiatica*	159,638	38.10	88,791	17,711	26,568	131	84	37	8	79,044	15 + 8
*A. glaucifolium*	160,400	37.90	80,251	17,637	31,256	139	94	36	8	84,105	16 + 8
*A. raddeana*	160,493	37.60	80,151	17,784	31,279	137	89	36	8	81,414	17 + 8
*A. macrophylla*	158,787	38.00	88,149	17,408	26,615	130	84	37	8	79,041	15 + 8
*A. coerulea*	161,429	39.00	91,119	17,354	26,478	140	81	45	8	75,984	12 + 8
*D. fargesii*	153,134	38.50	82,718	17,346	26,535	132	83	37	8	78,600	15 + 8
*L. fumarioides*	157,448	38.40	84,907	16,899	27,821	131	83	37	8	77,916	15 + 8
*P. anemonoides*	164,383	38.90	84,925	17,500	30,979	132	83	37	8	78,744	15 + 8

**Table 2:** List of genes annotated in the plastome of *N. sativa*.

Genes	Exon 1	Intron 1	Exon 2	Intron 2	Exon 3
*atp*F	145	723	410		
*clp*P	71	806	289	703	336
*ndh*A	553	985	539		
*ndh*B*	781	700	758		
*pet*B	5	775	644		
*pet*D	8	711	493		
*rpl*16	9	973	399		
*rpl*2*	391	659	434		
*rpo*C1	432	735	1602		
*rps*12*	114		232		26
*ycf*3	126	738	228	740	153

**Table 3:** The genes with introns in the plastome of *N. sativa* and the length of exons and introns.

Group of genes	Name of genes
Subunits of ATP synthase	*atp*A, *atp*B, *atp*E, *atp*F, *atp*H, *atp*I
Subunits of NADH-dehydrogenase	*ndh*A, *ndh*B, *ndh*C, *ndh*D, *ndh*E, *ndh*F, *ndh*G, *ndh*H, *ndh*I, *ndh*J, *ndh*K
Subunits of cytochrome b/f complex	*pet*A, *pet*B, *pet*D, *pet*G, *pet*L, *pet*N
Subunits of photosystem I	*psa*A, *psa*B, *psa*C, *psa*I, *psa*J
Subunits of photosystem II	*psb*A, *psb*B, *psb*C, *psb*D, *psb*E, *psb*F, *psb*H, *psb*I*, psbJ*, *psb*K, *psb*L, *psb*M, *psb*N, *psb*T, *psb*Z, *ycf*3
Large subunit of ribosome	*rpl*14, *rpl*16, *rpl*2*, *rpl*20, *rpl*22, *rpl*23*, *rpl*33, *rpl*36
Small subunit of the ribosome	*rps*11, *rps*12*, *rps*14, *rps*15, *rps*18, *rps*19, *rps*2, *rps*3, *rps*4, *rps*7*, *rps*8
DNA dependent RNA polymerase	*rpo*A, *rpo*B, *rpo*C1, *rpo*C2
Subunit of rubisco	*rbc*L
c-type cytochrome synthesis gene	*ccs*A
Envelop membrane protein	*cem*A
Maturase	*mat*K
Protease	*clp*P
Subunit of Acetyl-CoA-carboxylase	*acc*D
Translational initiation factor	*inf*A
Conserved open reading frames	*ycf*1, *ycf*2*, *yc*f4
Other genes	None

### Comparative analysis and divergence

**Figure 2:**
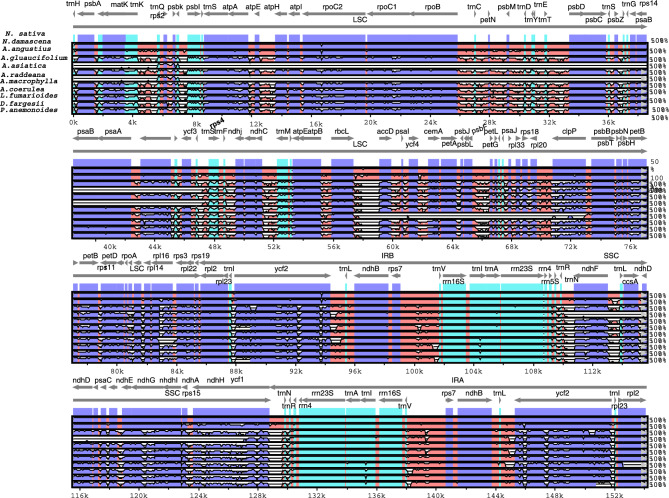
Alignment visualization of the *N. sativa* plastome sequences with related species. VISTA-based identity plot showing sequence identity among the 10 species using *N. sativa* as a reference. The vertical scale indicates percent identity, ranging from 50 to 100%. The horizontal axis indicates the coordinates within the plastome. Arrows indicate the annotated genes and their transcription direction. The thick black lines show the inverted repeats (IRs).

The mVISTA analysis uncovered sequence variability among 11 plastomes. In our results, the coding regions displayed comparatively low sequence divergence, while more significant divergence was observed in the non-coding regions. The results of the analysis revealed a noteworthy resemblance between *N. damascena* and *N. sativa* in comparison to other species. However, a distinctive pattern of divergence was observed in the region spanning from *trn*L to *ycf1*, particularly in the SSC region, as illustrated in Fig. 2. The analysis of various species revealed a variable number of divergences, with a notable pattern observed across different genomic regions. The most substantial divergences were identified within the LSC region, with *A. raddeana* and *A. glaucifolium*. Noteworthy divergences were also observed in other species, especially across the *psb*A to the *atp*H, *rpo*B to the *trn*T, and *yc*f3 to the *ndh*J regions. A striking divergence pattern was also evident in *A. coerulea*, exhibiting significant distinctions, especially within the *rbc*L to *clp*P region in the LSC position. In the SSC region, all plastomes exhibited pronounced divergences compared to *N. sativa* (Fig. 2). High divergence was noted from *ndh*F to *ycf1*, with *A. glaucifolium* showcasing a particularly significant divergence. Contrastingly, the IR region displayed relatively lower levels of divergence compared to the LSC and SSC regions. The *ycf*2 gene, however, demonstrated substantial divergence in the IR region across all species, with *P. anemonoides* exhibiting heightened distinctions. Furthermore, the *rpl*2 gene displayed notable divergence, particularly in *A. coerulea*.

**Figure 3:**
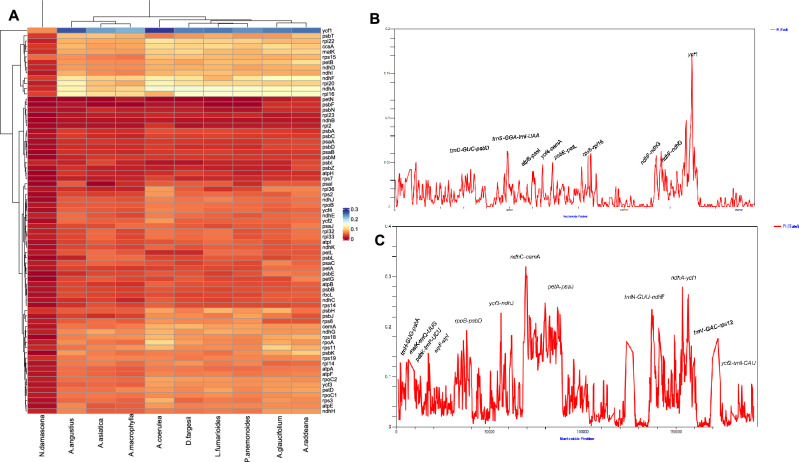
Pairwise sequence distance of 73 protein coding genes of *N. sativa* and related species (**A**). Nucleotide diversity (Pi) analysis for whole plastomes of *N. sativa* species. Sliding window length was 200 bp and step size was selected as 100 bp. X-axis: position of the midpoint of a window, Y-axis: nucleotide diversity (Pi) of each window. (**B**) Sliding window analysis of *N. sativa* and *N*. *damascena*. (**C**) Sliding window analyses of *N. sativa* with other 7 species.

The average pairwise sequence divergence was also calculated for the complete plastome and protein coding genes. *A. glaucifolium*’s plastome displayed the highest average pairwise sequence divergence (0.2851) with *N. sativa*, followed by *A. raddeana* (0.2290) and *A. coerulea* (0.1222). In contrast, *N. damascena* exhibited a low pairwise sequence divergence of 0.0117 with *N. sativa* (Table S1 and Fig. 3). Analysis of protein-coding gene divergence in selected plastomes reveals a distinct pattern, depicted in a heatmap. Notably, the *ycf*1 gene exhibits significant divergence compared to *N. sativa*, with other divergent genes including *rpl*14, *rpl*16, *rpl*20, *ccs*A*, cem*A*, mat*K*, psb*T*, ndh*A, and *ndh*F across all species, except *N. damascene*, which resembles *N. sativa*. The highest pairwise sequence divergence is observed in *ycf*1 at 0.2283. This study provides valuable insights into the evolutionary dynamics and genetic divergence among these species.

The complete plastome of *N. sativa* was aligned with *N. damascena*, and DnaSP software calculated nucleotide variability (Pi) to identify mutational hotspots. Nine highly variable loci with elevated Pi values were detected in the chloroplast genomes of both species, highlighting specific regions of sequence diversity. These include six divergent hotspots in LSC regions, *trnD-GUC-psb*D (0.055) and *trnS-GGA-trn*l*-UAA* (0.09), *atp*B*-psa*I (0.06), *ycf*4*-cem*A (0.08), *psb*E*-pet*L (0.065), *rps*8*-rpl*16 (0.1), and 3 in SSC region *ndh*F*-ndh*G (0.8), *ndh*I*-rps*15 (0.065), and *ycf*1 (0.21) (Fig. 3B). Our investigation involved a thorough multiple alignment of nine plastomes, excluding *A. glaucifolium* and *A. raddeana* due to their substantial divergence from *N. sativa*. The analysis revealed 12 divergent hotspot regions with Pi values exceeding 0.1. Noteworthy loci in the LSC region include *trn*H*-GUG-psb*A, *mat*K*-trn*Q*-UUG*, *atp*F*-atp*I, *rpo*B*-psb*D, *ycf*3*-ndh*J, *ndh*C*-cem*A, and *pet*A*-psa*J. In the IR region, *trn*N*-GUU-ndh*F, *trn*V*-GAC-rps*12, and *ycf*2*-trn*I*-CAU* exhibited divergence. In the SSC region, the *ndh*A*-ycf1* locus (0.27) stands out, as depicted in Fig. 3B. High Pi values in divergence regions highlight significant variations in the entire plastome of *N. sativa*. Specifically, the *ndh*C*-cem*A region shows the highest Pi value at 0.31, followed closely by *ndh*A*-ycf*1 at 0.27, providing insights into specific genomic distinctions in these areas.

### Plastomes structure variations, inversions, and divergence hotspots

**Figure 4:**
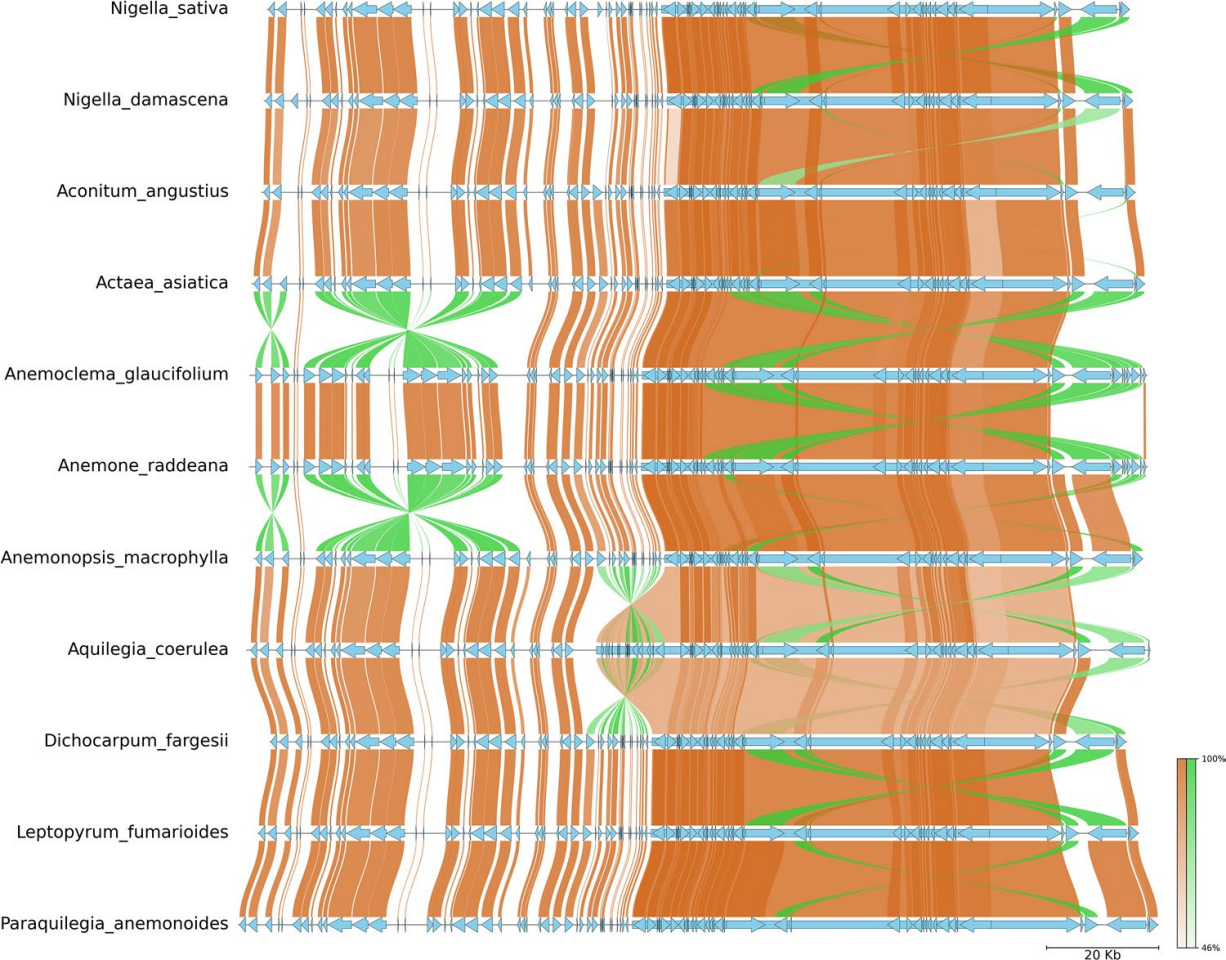
Synteny plot of *N. sativa* and ten other plastomes from Ranunculaceae family. The synteny plot shows normal links with chocolate color, inverted link with lime-green color, and gene feature with sky-blue color.

The plastome of the Ranunculaceae family is typically highly conserved, our study revealed variations in certain species compared to the *N. sativa* plastome. A significant 36 kb inversion in the LSC region (*ycf*3 to *atp*A genes) was identified in the plastomes of *A. raddeana* and *A. glaucifolium*. Additionally, a 19 kb inversion between *ycf*1 and *ndh*F genes in the SSC region was observed in the latter species (Fig. 4). Similarly, in the plastomes of *A. coerulea*, a 22 kb inversion from *atp*B to *clp*P in the large single-copy (LSC) region was observed (Fig. 4). *A. raddeana* and *A. glaucifolium* displayed minor inversions and shifts in the *psb*A and *trn*H*-GUG* to *psb*K region (LSC region). Rearrangements in *A. raddeana* and *A. glaucifolium* included the relocation of *trn*R*-UCU, trn*G*-UCC*, and *trn*S*-GCU* near *ndh*J*,* as well as the movement of *rps*4 and *rps*16 to the genome’s start. Notably, *trn*K*-UUU* and *mat*K shifted between *rps*16 and *psb*A genes. The absence of the *rps*16 gene in *N. sativa* and *A. angustius* was observed. Additionally, the *ycf*15 gene was exclusively present in *A. glaucifolium* (Fig. 4), highlighting distinct genomic variations and structural rearrangements in these chloroplast genomes.

### IR expansion and contraction

**Figure 5:**
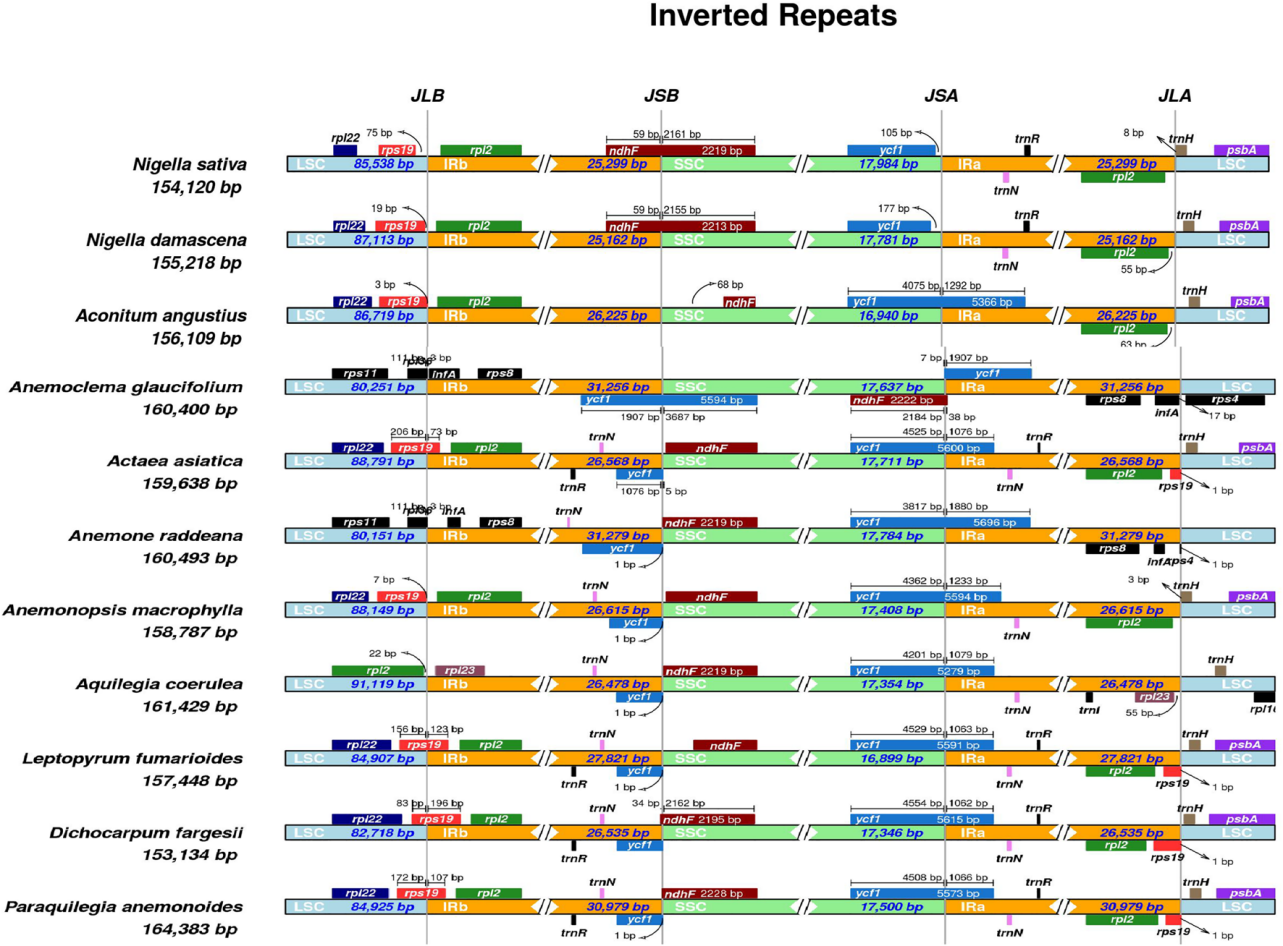
Comparison of junctions between the large single-copy (LSC), small single-copy (SSC) and inverted repeat (IR) regions among plastome of *N. sativa* and other ten plastomes. Boxes above or below the main line indicate the adjacent border genes. The numbers above the gene features indicate the distance between the ends of genes and border sites.

To explore the potential expansion and contraction of IRs, the distributions of IR and SC border regions in the plastomes of 11 taxa within the family Ranunculaceae were compared. The *rps*19 gene, present in all species except *A. raddeana*, *A. glaucifolium*, *P. anemonoides*, and *A. coerulea*, exhibited an unusual behavior by crossing the boundary between the LSC and IRb regions. Notably, the *rpl*22 gene consistently resided in the LSC region across species, except for *A. raddeana*, *A. glaucifolium*, and *A. coerulea*, where it was absent (Fig. 5). Additionally, the typical placement of the *rpl*2 gene in the IRb region shifted to the LSC region in *A. coerulea*. The *ycf*1 gene in *A. glaucifolium* fully overlaps the JSB boundary, while across all species, it spans the JSA boundary, predominantly in the IRa region. In *N. damascena* and *N. sativa*, *ycf*1 is in the SSC region. The *ndh*F gene is closer to the JSB boundary in all species except *A. glaucifolium*, where it extends beyond the JSA boundary. The *psb*A is absent in *A. glaucifolium*, *N. damascena*, and *N. sativa*. The *trn*H gene is absent in *A. raddeana* and *A. glaucifolium*. *A. coerulea* has the *rpl*23 gene in the IRb region, absent in other species. *A. raddeana* and *A. glaucifolium* exhibit unique gene arrangements, with *rps*11 in the LSC region, *inf*A in the IRb region in *A. raddeana*, and *rps*4 exclusively in *A. glaucifolium*’s LSC region (Fig. 5). This analysis highlights distinct plastome patterns among species. Structural variations in the IR and SSC regions can lead to gene rearrangements^40,41^. In this study, the lengths of IR regions were extended in *P. anemonoides* (30,979 bp), *A. glaucifolium* (31,256 bp), and *A. raddeana* (31,279 bp). This extension may contribute to the comparatively larger plastome sizes observed in these species compared to the IR region lengths of *N. sativa* (25,299 bp) and *N. damascena* (25,162 bp. Contraction and extension were identified in IR and SSC regions across all studied species. Additionally, in species such as *A. coerulea*, which has an extended plastome, there is an observable extension in the LSC region (Fig. 5).

### Repeat and SSR analysis

**Figure 6:**
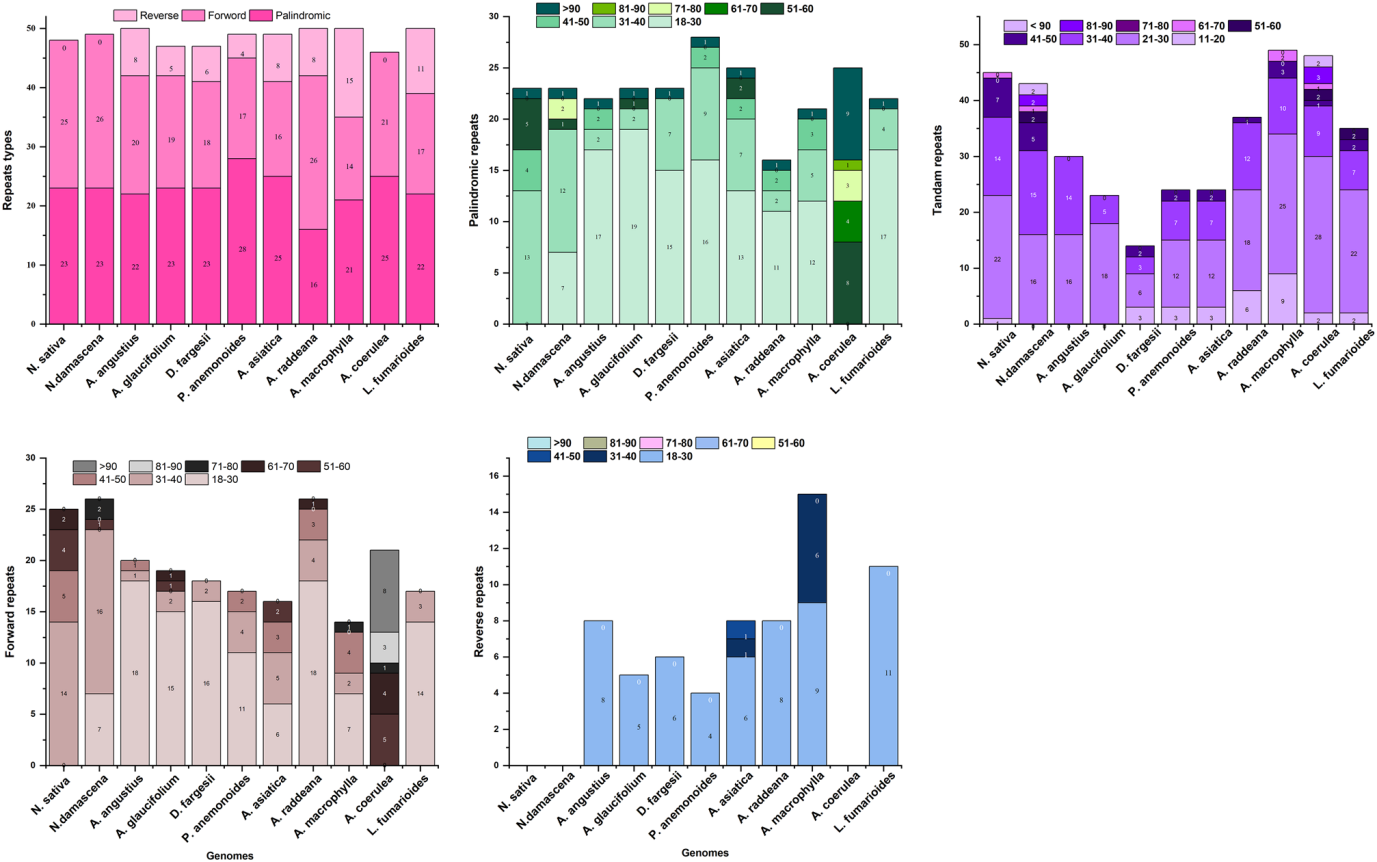
Analysis of repeated sequences in *N. sativa* and other 10 Ranunculaceae plastomes (**A**), totals numbers of three repeat types (**B**), number of palindromic repeats by length (**C**), number of tandem repeats by length (**D**), number of forward repeats by length (**E**) and number of reverse repeats by length.

**Figure 7:**
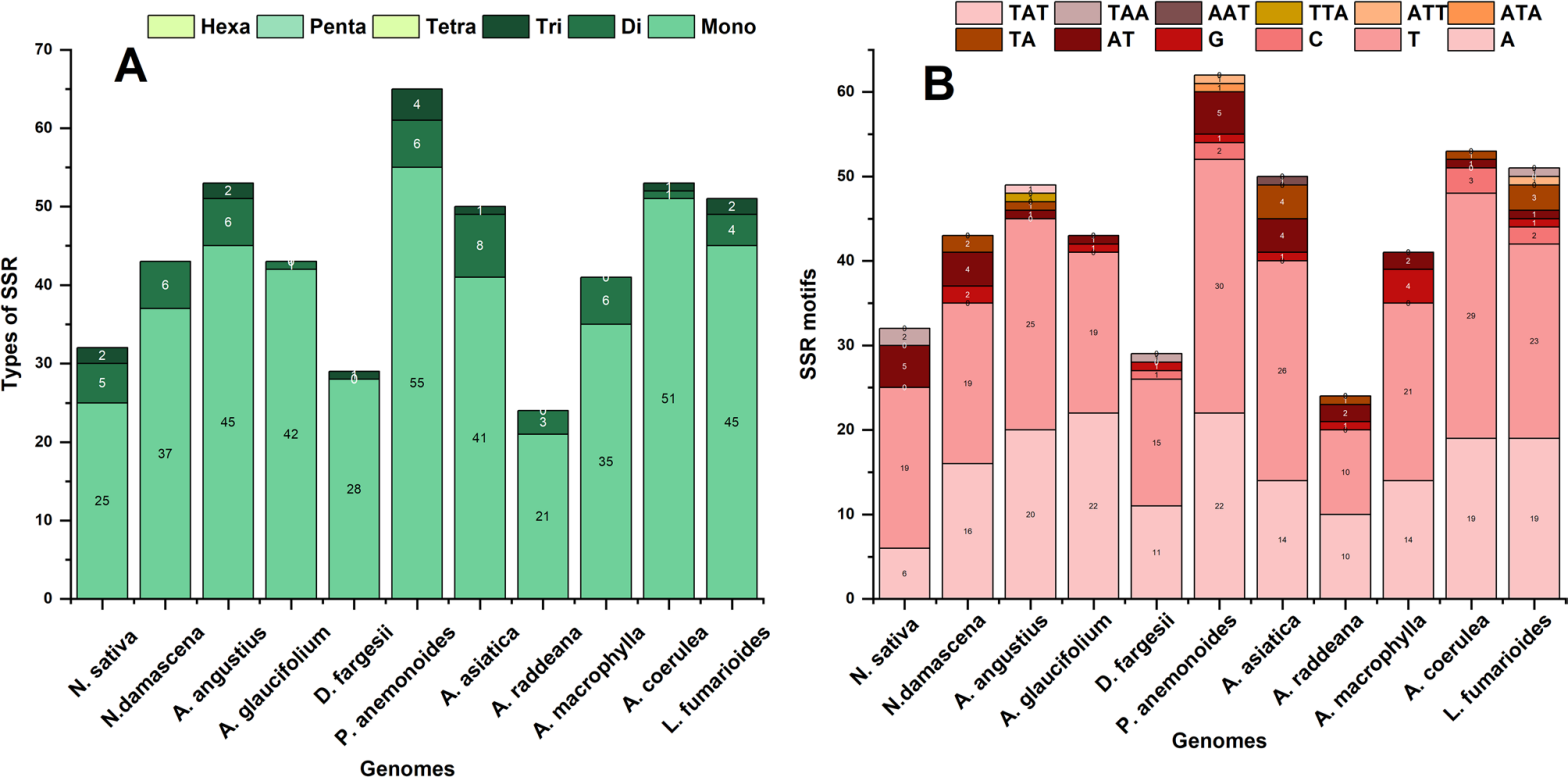
Number of different types of SSRs in the plastome of *N. sativa* and other plastomes (**A**) and number of SSR motifs (**B**).

The number of repeats identified in all selected species ranges from 46 to 50, encompassing 16 to 28 palindromic repeats, 17 to 26 forward repeats, and 0 to 15 reverse repeats (Fig. 6). In *N. sativa*, the total repeats are 48, including 23 palindromic repeats and 25 forward repeats, with no reverse repeats observed. Across the selected species, all repeat types are predominantly about 18–30 bp in length (Fig. 6). Tandem repeats vary from 14 to 49 in all species, most falling within the 11–20 bp range. Specifically, *N. sativa* exhibits 24 tandem repeats (Fig. 6C). The SSR analysis of 11 plastomes revealed diversity in microsatellite counts, notably, *N. sativa* displayed 32 repeats, predominantly consisting of mononucleotide repeats. Additionally, some di- and trinucleotide repeats are present in the SSR analysis. *P. anemonoides* exhibits the highest number of SSRs among all species, totaling 65 (Fig. 7A). The predominant type of SSRs across all plastomes were mononucleotide repeats, followed by dinucleotide and trinucleotide repeats. However, tetranucleotide, pentanucleotide, and hexanucleotide repeats were absent in all plastomes. A and T repeats constitute a more significant proportion of mononucleotide repeats than G and C repeats. Similarly, in dinucleotide repeats, the AT content represents a more significant proportion than the GC content (Fig. 7B).

### Phylogenetic analysis

**Figure 8:**
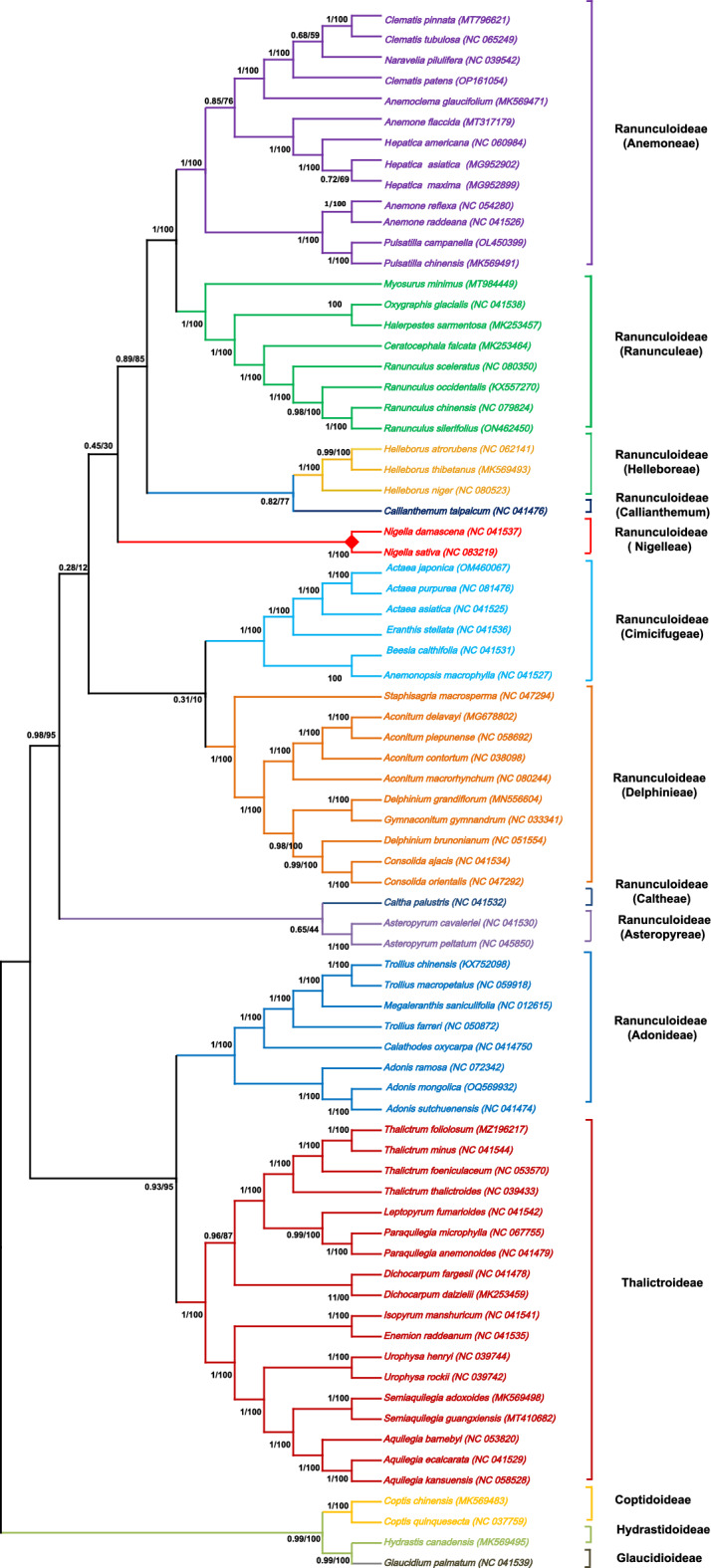
Phylogenetic trees were constructed for 75 members of the family Ranunculaceae, representing 11 different genera using different methods, and tree is shown for 73 commonly shared genes data sets constructed by Maximum Likelihood (ML) and Bayesian inference (BI) method. The number above on each node represents the bootstrap value. The red color diamond shape represents the position for *N. sativa*.

This study inferred phylogenetic relationships within Ranunculaceae from 73 shared protein coding genes. The Glaucidioideae, Hydrastidoideae, and Coptidoideae emerged as the earliest divergent lineages within the Ranunculaceae family in our study. In our current study, the analysis of plastid phylogenomics revealed a well-supported sister relationship between subfamilies Talictroideae and tribe Adonideae, with a strong bootstrap value of 95. The tribe Asteropyreae and Caltheae were observed to form the same clade in our study, but the support for this grouping is relatively low, with a bootstrap value of 44. Our analysis in Ranunculoideae successfully resolved the sister relationship between the tribes Anemoneae and Ranunculeae, with a robust bootstrap support value of 100 (Fig. 8). In our study, we observed that the position of Nigelleae is situated between Callianthemum and Cimicifugeae based on the protein coding genes data set. This tribe demonstrated its closest relationship with Cimicifugeae, a connection supported by a robust bootstrap value of 100. The phylogenetic trees strongly indicate that *N. sativa* is most closely related to *N. damascene*, which belongs to the genus *Nigella* and forms the same clade.

## Discussion

In recent years, the plastome has frequently been employed as a DNA super barcode for the identification, classification, and phylogenetic research of medicinal plants^42,43^. In this study, we utilized next-generation sequencing to sequence the first complete plastome of *N. sativa*. The observed quadripartite structure is consistent with the typical organization found in the majority of plastomes of land plants^22,44^. The plastome sizes exhibited a range, with *N. sativa* having a size of 154,120 bp and *P. anemonoides* displaying the largest size at 164,383 bp (Table 1). These findings align with previous studies indicating size variation among plastomes from different genera within the Ranunculaceae family. The plastome sizes in *Aquilegia, Delphinium,* and *Ranunculus* have been estimated at 151 kb, 149 kb, and 157 kb, respectively^45^. Earlier studies on different angiosperm groups have indicated that plastome can be conserved^46^ or highly polymorphic^47,48^. Currently, the comparison of 11 plastomes from various genera in the Ranunculaceae family has shown significant variation in plastome structure. Our research aligns with earlier research that found structural variation in *Clematis*, opposing the assumption of conserved characteristic structures in the plastome^49,50^. In the present study, we observed significant divergence in the plastome and gene order among genomes such as *A. raddeana*, *A. glaucifolium*, and *A. coerulea* compared to *N. sativa*. The most notable distinction from the plastome of *N. sativa* involved a substantial inversion of 36 kb. It was identified between *ycf*3 to *atp*A genes (LSC region) in the plastome of *A. raddeana* and *A. glaucifolium*, and another inversion of 19 kb was observed in between the *ycf*1 and *ndh*F genes (SSC region) in the latter species (Fig. 4). Similarly, an inversion of about 22 kb was detected in the plastome of *A. coerulea* between the *atp*B to *clp*P2 gene in the large single-copy (LSC) region. Furthermore, we observed several smaller inversions, shifts in genes, and rearrangements in the plastome of these species. However, the other species including *N. sativa* lake inversions and transpositions in their plastome. Our findings are consistent with the research conducted by^39^, indicating that *Clematis* has undergone four rearrangements compared to *Coptis*. *Coptis*, an ancestral condition in Ranunculaceae, exhibits a typical chloroplast structure. Similarly, minor changes were documented in the family Orchidaceae, specifically involving the inversion of the *petN-psbM* region^51^. In contrast, gymnosperms belonging to the Pinaceae family exhibited a distinct pattern with five different plastome structures^52^. The identification of inversion and transposition events in the plastome of *A. raddeana, A. coerulea*, and *A. glaucifolium* is consistent with prior research indicating that the occurrence of structural rearrangements in plastome varies within the family. Previous studies have reported the presence of inversions in genera such as *Anemone*, *Adonis*, and *Clematis*^53^. Besides, the work of^54^ is in line with our study that within Ranunculeae species, the plastome gene orders align with those of numerous other genera (e.g., *Aconitum, Thalictrum*), and no occurrences of gene inversions or translocations have been observed.

Plastome sequences among family Ranunculaceae species show significant genetic divergence, as documented in prior research^55^. Aligned sequences indicate substantial differentiation, particularly in noncoding regions and SSC and LSC regions. Nucleotide diversity (PI) shows the extent of variation in DNA sequences, providing insights into the genetic diversity within a species^56^. Nucleotide diversity (PI) values were higher in the chloroplast genes of *N. sativa* and its related species within the LSC and SSC regions compared to the IR region. This observation is consistent with findings in other angiosperms^57,58^. Our findings indicate that the plastome of *N. sativa* exhibits a high degree of sequence similarity with *N. damascena* species because both belong to the same genus. Nevertheless, there are regions where the identity is relatively lower in comparison. In contrast, the other nine plastomes display substantial sequence divergence from *N. sativa.* We compared the *N. sativa* plastome with seven other sequenced species, excluding *A. raddeana* and *A. glaucifolium,* due to their higher divergence. Through sliding window analysis, we identified 12 divergent hotspot regions, including *trn*H*-GUG-psb*A (0.12), *mat*K*-trn*Q*-UUG* (0.13), *psb*K*-trn*R*-UCU* (0.1), *atp*F-*atp*I (0.12), *rpo*B*-psb*D (0.19), *ycf*3*-ndh*J (0.22), *ndh*C*-cem*A (0.31), *pet*A*-psa*J (0.24), *trn*N*-GUU-ndh*F (0.23), *trn*V*-GAC-rps*12 (0.17), and *ycf*2*-trn*I*-CAU* (0.092) and *ndh*A*-ycf1* (0.27). The significantly divergent regions identified here offer valuable insights for developing molecular markers in plant identification and exploring phylogenetic relationships of *N. sativa* and related species. The detection of these positively selected sites such as *atp*F-*atp*I, rpoB*-psb*D, *yc*f3-*ndh*J, *ndh*C-*cem*A, and *pet*A-*psa*J suggests that these regions have undergone adaptations to environmental stressors^59^. The identification and classification of Ranunculaceae species are crucial for understanding their evolutionary relationships and ecological roles^59,60^. The previous research revealed that the combination of markers such as *ndh*C-*trn*V-UAC, *psb*E-*pet*L, *rps*8-*rpl*14, *pet*N-*psb*M, *atp*F-*atp*I, *trn*T-GGU-*psb*D, r*pl*32-*trn*L-UAG, *rpl*16-*rps*3, *rps*16-*trn*Q-UUG, *ndh*G-*ndh*I, *acc*D-*psa*I, *trn*G-GCC-*trn*fM-CAU, *trn*T-UGU-*trn*L-UAA, *psb*Z-*trn*G-GCC, and *trn*K-UUU-rps16 resulted in a 100% species identification rate, which is significantly higher than the rates achieved by individual markers^59–62^. The study also revealed that the use of combination markers can identify seven-fold more variant sites than conventional single-specific barcode markers Kim et al. This observation aligns with previous findings in the Ranunculaceae family, where over 20 divergent hotspot regions were identified^59^. Similarly, nine divergent hotspot regions in seven species of *Pulsatilla* (Ranunculaceae) were identified previously, including six intergenic spacer regions (*rps4-rps16, rps16-mat*K*, ndh*C*-trn*V*, psb*E*-pet*L*, ndh*D*-ccs*A and *ccs*A*-ndh*F) and four protein-coding regions (*ycf*1, *ndh*F and *ndh*I)^60^. These findings underscore the value of using multiple markers to account for the varying rates of nucleotide variation across different loci. The use of these combined markers can be particularly advantageous for identifying closely related species, where individual markers may not be sufficient to distinguish between them. The most effective multi-locus barcode for identifying *Pulsatilla* species from the Ranunculaceae family was found to be cpDNA barcodes like *rbc*L, *mat*K and *trn*H-psbA in earlier research^60^. Furthermore, *ycf*1 gene was also found the most efficient barcode in *Aconitum* species identification^61^.

Additionally, our findings indicate that Angiosperms tend to accumulate variations at the genus level in the LSC and SSC regions of the plastome. This pattern is consistent with the distribution of variations reported in the plastomes of other genera, such as *Cymbidium*, *Oenothera*, and *Pyrus*^63^. Moreover, the observed distribution of divergence regions, predominantly in the LSC and SSC regions, aligns with previous reports on Chaenomeles and Lancea species^64,65^. Previously, five types of plastome were identified based on distinctions in the LSC region*. N. damascena* (Type I) represents an ancestral condition. *A. raddeana* and *A. glaucifolium* exhibit the second type (Type II) with a unique gene arrangement pattern involving inversions. Likewise, *A. coerulea* (Type V) features an inversion between *accD* and *clp*P1, distinguishing it from Type I chloroplast genomes. In the Ranunculaceae, the Type I plastome is considered the most primitive. According to^39^, all other types have originated from Type I through the inversion of different genes.

The concept of codon usage bias (CUB) refers to the differential frequency with which various synonymous codons encoding the same amino acid are observed in the coding sequences of a given organism’s genome^48^. CUB preferences are specific to different genes in different species and can even vary within a particular species. This variability is shaped by a combination of factors, including mutation, selection, and genetic drift, which act during the long-term evolution of genes and species^66^. In our study, we examined the codon usage frequency of protein-coding genes in the *N. sativa* plastome, among all phenylalanine had the highest codons (1982). Additionally, 35 codons analyzed exhibited a relative synonymous codon usage (RSCU) greater than 1 while the most favored codon was AGA, encoding arginine, with an RSCU value of 1.78.

The plastome of higher plants is known for its high degree of conservation. However, variations in genome length between species do arise due to the dynamic processes of extension and contraction occurring in the IR, LSC, and SSC regions^67–71^. Throughout plastome evolution, the IR region undergoes dynamic changes involving expansion and contraction, with genes entering either the IR region or the LSC and SSC regions^72^. We thoroughly compared 11 species, examining the two IRs and the two single-copy regions. In *N. sativa*, a notable contraction was observed in the IRs, while only a slight expansion was noted in the SSC region due to the shifting of *rpl2* and *ycf1* genes, leading to a shortened plastome length (Fig. 7). On the contrary, in *P. anemonoides*, there is an extension in the IR region. The larger genome size of this species might be due to the *rps19* gene entering the junction of the LSC and IR borders, and 107 bp appeared in the IR region and was duplicated. Similarly, *A. raddeana* and *A. glaucifolium* exhibit expanded IR regions with placed genes *inf*A, *rps*8*, rpl*2*, ycf*1, and *rpl*36 extending to the JLB Junction. Additionally, *rps*11 and *rps*4 genes are situated in the LSC region, contributing to increased genome size. The expanded genome size in *A. coerulea* results from LSC region enlargement, while SSC and IR regions simultaneously contract. This aligns with previous research indicating significant structural changes in land plant plastomes, including IR region loss or specific gene families^73^. The events of expansion and contraction in IRs are crucial in evolution as they can lead to alterations in gene content and plastome size^47,74^. The expansion of IRs has been documented in Araceae^74,75^. In certain cases, the LSC region expands while the SSC region decreases, reaching a size of only 7000 bp in Pothos^76^. The expansion and contraction of IR regions can result in the duplication or conversion of certain genes from duplicate to a single copy, respectively^47,74^. Modifications in IR size can also prompt rearrangements of genes in the SSC region, as recently observed in Zantedeschia^74^.

Long repeats are crucial contributors to the complete plastome’s variation, expansion, and rearrangement^77^. *N. sativa* was found to have approximately 48 long repeats. In comparison, the long repeats in these plastomes ranged from 46 (*A. coerulea*) to 50 (*A. raddeana, A. macrophylla, A. angustius*). The SSRs and long repeats in the 11 plastomes showed considerable variation. SSRs were mainly present in the non-coding region, and their sequence variation was higher compared to the coding region^78^. Additionally, SSRs can be employed for studying conservation genetics in endangered plant species, molecular identification, and exploring genetic relationships among related species^79,80^. The analysis of SSRs in the plastome of *N. sativa* revealed variations in the number of SSRs among 11 species, ranging from 24 (*A. raddeana*) to 65 (*P. anemonoides*). Mononucleotide repeats are the most common, followed by dinucleotide repeats, and the prevalent motifs across all species are A and T. Our results align with previous reports indicating that mononucleotide and dinucleotide repeats were the most and second most abundant SSRs in the plastomes of two *Caldesia* species^81^. Additionally, our findings are in line with earlier research suggesting that SSRs in plastome predominantly consist of polythymine (polyT) or polyadenine (polyA) repeats and less frequently contain tandem cytosine (C) and guanine (G) repeats^82^. This consistency supports the previous observation that plastome SSRs are primarily dominated by ‘A’ or ‘T’ mononucleotide repeats^83,84^.

The current classification of Ranunculaceae, as proposed by^85^, relies on a comprehensive analysis that combines both morphological and molecular phylogenetic data. This classification results from examining 6957 molecular characters and 65 morphological characters. In this proposed classification, Ranunculaceae is categorized into five monophyletic subfamilies: Glaucidioideae, Hydrastidoideae, Coptidoideae, Thalictroideae, and Ranunculoideae. The Ranunculoideae subfamily is further subdivided into ten strongly supported monophyletic tribes. The findings of our study align with previous research, supporting Glaucidium as the first diverging taxon and sister to all other Ranunculaceae species^85–87^. Our results are consistent with the findings of^85^, indicating that Hydrastis is the second diverging taxon with robust support, and Coptidoideae represents the third diverging clade. In earlier studies, the position of Nigelleae within the Ranunculaceae family has been inconsistent. However, a previous analysis of plastomes from 38 Ranunculaceae species found that Nigelleae is closely related to Delphineae. This relationship was strongly supported by a bootstrap value (100), providing robust evidence for the clustering of Nigelleae and Delphineae in the same clade^88^. Furthermore, based on 77 protein-coding genes and four rRNA genes, the analysis revealed that Caltheae is the sister group to Asteropyreae. In turn, Asteropyreae is identified as the sister group to the combined clade of Caltheae, Delphinieae, and Nigelleae^39^. Nevertheless, our findings align with the research conducted by^89,90^, where they identified Nigellaea as the sister group to Cimicifugeae. Similar results about Nigelleae were reported previously^91^. Furthermore, in line with our study, they also identified the sister relationship between the subfamilies Talictroideae and Adonideae. Moreover, in our research, the strongest supported grouping (with a bootstrap value of 100) among tribes of Ranunculoideae is the sister group relationship between Anemoneae and Ranunculeae. This finding is consistent with results from previous studies, providing additional confirmation to the observed relationship between these two tribes^85,92–94^. The data obtained from our study offers valuable insights for future genetic and evolutionary investigations of *N. sativa* and the broader Ranunculaceae family.

## Conclusions

In conclusion, the sequencing and comparative analysis of the complete plastome of *N. sativa* were conducted for the first time, and the results were compared with those of other related species. The comparison highlighted the conservation of the overall structure in the available complete plastome of *N. sativa*. However, notable variations were observed in gene order, and certain structural changes were identified, primarily caused by the expansion or contraction of the IR regions into or out of adjacent single-copy regions. The comparative analysis of plastome *N. sativa* and other studied plants unveiled highly variable regions, including *trn*H*-GUG-psb*A*, mat*K*-trn*Q*-UUG, psb*K*-trn*R*-UCU, atp*F*-atp*I*, rpo*B*-psb*D*, ycf*3*-ndh*J*, ndh*C*-cem*A*, pet*A*-psa*J*, trn*N*-GUU, ndh*F*, trn*V*-GAC-rps*12, and *ycf*2*-trn*I*-CAU*. These regions are identified as fast-evolving loci and show promise as molecular markers in future studies. SSRs and long repeat sequences were identified in terms of number and types, providing potential and effective options for developing molecular markers. The phylogenetic analysis showed that *N. sativa* forms the same clade as *N. damascene* with a high bs value (100). However, this tribe is a successive sister to the Cimicifugeae tribe with strong support. The thorough analysis of these complete plastomes contributes valuable insights to conserving medicinal resources, understanding genetic diversity, exploring genome evolution and adaptation history, and investigating the phylogenetic relationships of *N. sativa* plants.

## Materials and methods

The fresh leaves were collected from *N. sativa* cultivate in Agriculture Research Center, KPK, Pakistan and transported in liquid nitrogen to the − 80 °C facility. The specimens were submitted to the Agriculture Research Center KP, Pakistan herbarium center under the voucher numbers AGN-NG1 (*N. sativa*). Dr. Muhammad Waqas one of the leading agronomists at the Agriculture Research Center KPK, Pakistan, identified the plants. The plant samples were collected and processed per the national guidelines and legislation. Hence, a permission permits (NJ334/15/78) was obtained from the Environmental Protection Agency, Khyber Pakhtunkhwa, Pakistan.

### DNA extraction and sequencing

To extract high-quality DNA from young and immature leaves of *N. sativa*, we employed a meticulous process. Firstly, the leaves were finely ground into a fine powder using liquid nitrogen. This method ensured that the DNA would be released from the cells effectively. To isolate the DNA, we utilized the highly reliable DNeasy Plant Mini Kit from Qiagen (Valencia, CA, USA). This kit provided us with a robust and efficient method for DNA extraction from plant samples. The kit's protocol was followed carefully to obtain high-quality DNA. Once the DNA was successfully isolated, we proceeded to sequence the chloroplast DNA using an Illumina HiSeq-2000 platform at Macrogen (Seoul, Korea). This cutting-edge sequencing platform allowed us to generate a vast number of raw reads for *N. sativa*, specifically around 578,630,881 raw reads. However, to ensure the reliability and accuracy of our analysis, we needed to filter out low-quality sequences. To achieve this, we implemented a stringent filtering criterion based on a Phred score of less than 30. This quality control step eliminated any reads that did not meet the desired threshold, ensuring that only high-quality sequences were retained for further analysis. To assemble the plastome with precision, we employed two different methods. Firstly, we utilized the GetOrganelle v 1.7.5 pipeline^95^, which is a sophisticated tool specifically designed for plastome assembly. Additionally, we also employed SPAdes version 3.10.1 (http://bioinf.spbau.ru/spades) as an assembler to enhance the accuracy and reliability of the assembly process.

### Genome annotation

The annotation process of the plastome involved several steps using established tools and software. CpGAVAS2^96^ and GeSeq (https://chlorobox.mpimp-golm.mpg.de/geseq.html), widely recognized online tools for genome annotation, were utilized to carry out the initial annotation. Additionally, tRNAscan-SE^97^, a well-established program, was employed to identify tRNA genes within the plastomes. To ensure the accuracy of the annotations, a comparative analysis was conducted by comparing the plastomes with reference genomes using Geneious Pro v.10.2.3^98^ and tRNAs can-SE (v.1.21)^97^. This step allowed for the identification of start and stop codons, determination of intron boundaries, and implementation of manual alterations when necessary. To visualize the structural features of the plastomes, chloroplot, a powerful tool^99^, was used. Furthermore, the genomic divergence was assessed using mVISTA in shuffle-LAGAN mode, with the plastome of *N. sativa* serving as the reference^55^. In the *N. sativa* plastome, the average pairwise sequence divergence with ten related species (*N. damascena*, *A. asiatica, A. angustius, A. raddeana, A. coerulea*, *A. glaucifolium, P. anemonoides, L. fumarioides, D. fargesii* and *A. macrophylla*) was determined. We extensively compared gene order and performed multiple sequence alignment. This allowed us to employ comparative sequence analysis to identify any missing or unclear gene annotations. For whole genome alignment, we used MAFFT version 7.222 with default parameters^100^. Pairwise sequence divergence was calculated using Kimura’s two-parameter (K2P) model. This approach ensured an accurate assessment of the genetic data. In our analysis, we employed the DnaSP software version 6.13.03^101^ to perform a sliding window analysis with a window size of 200 bp and a step size of 100 bp. This analysis allowed us to calculate nucleotide variations, specifically the nucleotide diversity (Pi). To visualize the shared genes and gene divergence among different species plastomes, we utilized the heatmap2 package in the R software. Additionally, we created a synteny plot using the pyGenomeViz version 0.2.1 package, employing the pgv-mmseqs mode and setting an identity threshold of 50%. The relevant source for pyGenomeViz can be found on GitHub at the following URL: https://github.com/moshi4/pyGenomeViz.

### Characterization of repetitive sequences and SSRs

We identified various functional repetitive sequences within the plastomes of *N. sativa* and 10 other species belonging to the Ranunculaceae family. We identified palindromic, forward, and reverse repeat sequences using the online tool REPuter^102^. The analysis was conducted with conditions specifying a minimum repeat size of 8 base pairs and a maximum of 50 computed repeats. Likewise, the MISA software^103^ was employed to calculate simple sequence repeats (SSRs) under specific conditions: ≥ 8 repeat units for one base pair repeats, ≥ 6 repeat units for two base pair repeats, ≥ 4 repeat units for 3 and 4 base pair repeats, and ≥ three repeat units for 5 and 6 base pair repeats. Moreover, tandem repeats were computed using the online tool Tandem Repeats Finder v.4.09^104^.

### Genome divergence

We assessed the variation in shared protein-coding genes and complete plastomes among *N. sativa* and its related species. A comparative analysis was executed through multiple sequence alignment, wherein the examination and analysis of gene order were undertaken to enhance the precision of deficient and ambiguous gene annotations. Plastome annotations were conducted using MAFFT version 7.222^100^, employing default parameters. Pairwise sequence divergence was calculated utilizing Kimura’s two-parameter model (K2P)^100^. We created a synteny plot using the pyGenomeViz version 0.2.1 package, employing the pgv-mmseqs mode and setting an identity threshold of 50%. The relevant source for pyGenomeViz can be found on GitHub at the following URL: https://github.com/moshi4/pyGenomeViz.

### Phylogenetic analyses

To determine the phylogenetic position of *N. sativa* within the family Ranunculaceae, 76 published plastome sequences of Ranunculaceae species were downloaded from the NCBI database for phylogenetic analysis. A comprehensive analysis was conducted using a dataset comprising 73 commonly shared genes among 75 members of the family Ranunculaceae, representing 11 different genera. To ensure accuracy, the nucleotide sequences of these 73 protein-coding genes were aligned and combined using MAFFT, employing the default settings as outlined by^105^. The best-fitting model of nucleotide evolution, TVM + F + I + G4, was determined by jModelTest 2^106^. Two distinct approaches were employed to deduce the phylogenetic relationship of *N. sativa*. Firstly, a Bayesian inference (BI) tree was constructed using Mrbayes 3.12, utilizing the Markov chain Monte Carlo sampling method. Secondly, a maximum likelihood (ML) tree was generated using PAUP* 4.0^107^. The ML tree was created by running 1000 bootstraps, which provided support values for different nodes. For the BI analysis, a total of four chains were employed: three heated chains and one cold chain. These chains were run for 10,000,000 generations, with a sampling frequency of 1000 and a print frequency of 10,000. To ensure convergence, a burn-in of 2500 (25% of the total number of generations divided by the sampling frequency) was implemented. Finally, a 50% majority-rule consensus tree was derived from the phylogenetic trees generated, and Figtree^108^ was utilized to visually represent the relationships among the moss species based on their plastome sequences.

### Ethics approval and consent to participate

The authors declared that experimental research works on the plant described in this paper comply with institutional, national, and international guidelines. Field studies were conducted in accordance with local legislation and got permission from the provincial department of Forest and Grass of Khyber Pakhtunkhwa Province, Pakistan.

### Supplementary Information

Supplementary Table S1.

## Data Availability

All data generated or analyzed during this study are included in this published article. *N. sativa* plastome was submitted to NCBI with accession number (OR473632).

## References

[1] Park I (2017). The complete chloroplast genome sequence of *Aconitum*
*coreanum* and *Aconitum*
*carmichaelii* and comparative analysis with other Aconitum species. PLoS One.

[2] Shaw J, Lickey EB, Schilling EE, Small RL (2007). Comparison of whole chloroplast genome sequences to choose noncoding regions for phylogenetic studies in angiosperms: The tortoise and the hare III. Am. J. Bot..

[3] Mardanov AV (2008). Complete sequence of the duckweed (*Lemna*
*minor*) chloroplast genome: Structural organization and phylogenetic relationships to other angiosperms. J. Mol. Evol..

[4] Moore MJ, Soltis PS, Bell CD, Burleigh JG, Soltis DE (2010). Phylogenetic analysis of 83 plastid genes further resolves the early diversification of eudicots. Proc. Natl. Acad. Sci..

[5] Sun M, Li J, Li D, Shi L (2017). Complete chloroplast genome sequence of the medical fern *Drynaria*
*roosii* and its phylogenetic analysis. Mitochondrial DNA Part B.

[6] Asaf S, Ahmad W, Al-Harrasi A, Khan AL (2022). Uncovering the first complete plastome genomics, comparative analyses, and phylogenetic dispositions of endemic medicinal plant *Ziziphus*
*hajarensis* (Rhamnaceae). BMC Genom..

[7] Jansen RK (2005). Methods in Enzymology.

[8] Walker JF, Zanis MJ, Emery NC (2014). Comparative analysis of complete chloroplast genome sequence and inversion variation in *Lasthenia*
*burkei* (Madieae, Asteraceae). Am. J. Bot..

[9] Doyle JJ, Doyle JL, Ballenger J, Palmer J (1996). The distribution and phylogenetic significance of a 50-kb chloroplast DNA inversion in the flowering plant family Leguminosae. Mol. Phylogenet. Evol..

[10] Tangphatsornruang S (2011). Characterization of the complete chloroplast genome of *Hevea*
*brasiliensis* reveals genome rearrangement, RNA editing sites and phylogenetic relationships. Gene.

[11] Walker JF, Jansen RK, Zanis MJ, Emery NC (2015). Sources of inversion variation in the small single copy (SSC) region of chloroplast genomes. Am. J. Bot..

[12] Palmer JD, Nugent JM, Herbon LA (1987). Unusual structure of geranium chloroplast DNA: A triple-sized inverted repeat, extensive gene duplications, multiple inversions, and two repeat families. Proc. Natl. Acad. Sci..

[13] Tangphatsornruang S (2010). The chloroplast genome sequence of mungbean (*Vigna*
*radiata*) determined by high-throughput pyrosequencing: Structural organization and phylogenetic relationships. DNA Res..

[14] Fullerton SM, Bernardo Carvalho A, Clark AG (2001). Local rates of recombination are positively correlated with GC content in the human genome. Mol. Biol. Evol..

[15] Smith NG, Webster MT, Ellegren H (2002). Deterministic mutation rate variation in the human genome. Genome Res..

[16] Hiratsuka J (1989). The complete sequence of the rice (*Oryza*
*sativa*) chloroplast genome: Intermolecular recombination between distinct tRNA genes accounts for a major plastid DNA inversion during the evolution of the cereals. Mol. Gen. Genet. MGG.

[17] Johansson JT (1999). There large inversions in the chloroplast genomes and one loss of the chloroplast gene rps 16 suggest an early evolutionary split in the genus *Adonis* (Ranunculaceae). Plant Syst. Evol..

[18] Jansen RK, Wojciechowski MF, Sanniyasi E, Lee S-B, Daniell H (2008). Complete plastid genome sequence of the chickpea (*Cicer*
*arietinum*) and the phylogenetic distribution of rps12 and clpP intron losses among legumes (Leguminosae). Mol. Phylogenet. Evol..

[19] Yan M, Moore MJ, Meng A, Yao X, Wang H (2017). The first complete plastome sequence of the basal asterid family Styracaceae (Ericales) reveals a large inversion. Plant Syst. Evol..

[20] Tamura M (1993). Flowering Plants· Dicotyledons: Magnoliid, Hamamelid and Caryophyllid Families.

[21] Ro K-E, Keener CS, McPheron BA (1997). Molecular phylogenetic study of the Ranunculaceae: Utility of the nuclear 26S ribosomal DNA in inferring intrafamilial relationships. Mol. Phylogenet. Evol..

[22] Liu H (2018). Comparative analysis of complete chloroplast genomes of Anemoclema, Anemone, Pulsatilla, and Hepatica revealing structural variations among genera in tribe Anemoneae (Ranunculaceae). Front. Plant Sci..

[23] The Angiosperm Phylogeny Group (2009). An update of the Angiosperm Phylogeny Group classification for the orders and families of flowering plants: APG III. Bot. J. Linn. Soc..

[24] Compton JA, Culham A, Jury SL (1998). Reclassification of Actaea to include Cimicifuga and Souliea (Ranunculaceae): Phytogeny inferred from morphology, nrDNA ITS, and cpDNA trnL-F sequence variation. Taxon.

[25] Miikeda O, Kita K, Handa T, Yukawa T (2006). Phylogenetic relationships of Clematis (Ranunculaceae) based on chloroplast and nuclear DNA sequences. Bot. J. Linn. Soc..

[26] Falck D, Lehtonen S (2014). Two new names in Clematis (Ranunculaceae). Phytotaxa.

[27] Jiang N (2017). Phylogenetic reassessment of tribe Anemoneae (Ranunculaceae): Non-monophyly of Anemone sl revealed by plastid datasets. PLoS One.

[28] Compton JA, Hedderson TA (1997). A morphometric analysis of the *Cimicifuga*
*foetida* L. complex (Ranunculaceae). Bot. J. Linn. Soc..

[29] Zohary M (1983). The genus Nigella (Ranunculaceae)—A taxonomic revision. Plant Syst. Evol..

[30] Dönmez AA, Aydin ZU, Dönmez EO (2021). Taxonomic monograph of the tribe Nigelleae (Ranunculaceae): A group including ancient medicinal plants. Turk. J. Bot..

[31] Tutin T, Akeroyd J (1964). Nigella. Flora Europaea.

[32] Raab-Straube, E. V., Hand, R., Hörandl, E. & Nardi, E. Ranunculaceae. *Euro+ Med Plantbase–the information resource for Euro-Mediterranean plant diversity. *http://ww2.bgbm.org/EuroPlusMed/ (Accessed December 10, 2020) (2014).

[33] Ghosh A, Datta AK (2006). Karyotyping of *Nigella*
*sativa* L. (black cumin) and *Nigella*
*damascena* L. (love-in-a-mist) by image analyzing system. Cytologia.

[34] Malhotra S (2012). Handbook of Herbs and Spices.

[35] Shaker SS, Mohammadi A, Shahli MK (2017). Cytological studies on some ecotypes of *Nigella*
*sativa* L. in Iran. Cytologia.

[36] Birhanu K, Tileye F, Yohannes P, Said M (2015). Molecular diversity study of black cumin (*Nigella*
*sativa* L.) from Ethiopia as revealed by inter simple sequence repeat (ISSR) markers. Afr. J. Biotechnol..

[37] Mirzaei K, Mirzaghaderi G (2017). Genetic diversity analysis of Iranian *Nigella*
*sativa* L. landraces using SCoT markers and evaluation of adjusted polymorphism information content. Plant Genet. Resour..

[38] Sun Y (2017). Complete plastome sequencing of both living species of Circaeasteraceae (Ranunculales) reveals unusual rearrangements and the loss of the ndh gene family. BMC Genomics.

[39] Zhai W (2019). Chloroplast genomic data provide new and robust insights into the phylogeny and evolution of the Ranunculaceae. Mol. Phylogenet. Evol..

[40] Hirao T, Watanabe A, Kurita M, Kondo T, Takata K (2008). Complete nucleotide sequence of the *Cryptomeria*
*japonica* D. Don chloroplast genome and comparative chloroplast genomics: Diversified genomic structure of coniferous species. BMC Plant Biol..

[41] Zeng S (2017). The complete chloroplast genome sequences of six *Rehmannia* species. Genes.

[42] Rønsted N, Law S, Thornton H, Fay MF, Chase MW (2005). Molecular phylogenetic evidence for the monophyly of Fritillaria and Lilium (Liliaceae; Liliales) and the infrageneric classification of Fritillaria. Mol. Phylogenet. Evol..

[43] Xia C, Wang M, Guan Y, Li J (2022). Comparative analysis of the chloroplast genome for *Aconitum* species: Genome structure and phylogenetic relationships. Front. Genet..

[44] Tang Y, Yukawa T, Bateman RM, Jiang H, Peng H (2015). Phylogeny and classification of the East Asian Amitostigma alliance (Orchidaceae: Orchideae) based on six DNA markers. BMC Evol> Biol..

[45] Palmer JD (1991). Plastid chromosomes: Structure and evolution. Mol. Biol. Plastids.

[46] Henriquez CL (2020). Molecular evolution of chloroplast genomes in Monsteroideae (Araceae). Planta.

[47] Mehmood F (2020). Chloroplast genome of Hibiscus rosa-sinensis (Malvaceae): Comparative analyses and identification of mutational hotspots. Genomics.

[48] Qian W, Yang J-R, Pearson NM, Maclean C, Zhang J (2012). Balanced codon usage optimizes eukaryotic translational efficiency. PLoS Genet..

[49] Park S, An B, Park S (2020). Recurrent gene duplication in the angiosperm tribe Delphinieae (Ranunculaceae) inferred from intracellular gene transfer events and heteroplasmic mutations in the plastid matK gene. Sci. Rep..

[50] Sinn BT, Sedmak DD, Kelly LM, Freudenstein JV (2018). Total duplication of the small single copy region in the angiosperm plastome: Rearrangement and inverted repeat instability in Asarum. Am. J. Bot..

[51] Yang J-B, Tang M, Li H-T, Zhang Z-R, Li D-Z (2013). Complete chloroplast genome of the genus *Cymbidium*: Lights into the species identification, phylogenetic implications and population genetic analyses. BMC Evol. Biol..

[52] Wu C-S, Wang Y-N, Hsu C-Y, Lin C-P, Chaw S-M (2011). Loss of different inverted repeat copies from the chloroplast genomes of Pinaceae and cupressophytes and influence of heterotachy on the evaluation of gymnosperm phylogeny. Genome Biol. Evol..

[53] Hoot SB, Palmer JD (1994). Structural rearrangements, including parallel inversions, within the chloroplast genome of Anemone and related genera. J. Mol. Evol..

[54] Ji J (2023). Complete plastid genomes of nine species of Ranunculeae (Ranunculaceae) and their phylogenetic inferences. Genes.

[55] Frazer KA, Pachter L, Poliakov A, Rubin EM, Dubchak I (2004). VISTA: Computational tools for comparative genomics. Nucleic Acids Res..

[56] Akhunov ED (2010). Nucleotide diversity maps reveal variation in diversity among wheat genomes and chromosomes. BMC Genomics.

[57] Wang C (2021). Complete chloroplast genome sequence of *Sonchus*
*brachyotus* helps to elucidate evolutionary relationships with related species of Asteraceae. BioMed Res. Int..

[58] Zhang Y (2021). Complete chloroplast genome analysis of two important medicinal *Alpinia* species: *Alpinia*
*galanga* and *Alpinia*
*kwangsiensis*. Front. Plant Sci..

[59] Kim K-R (2023). Complete chloroplast genome determination of *Ranunculus*
*sceleratus* from Republic of Korea (Ranunculaceae) and comparative chloroplast genomes of the members of the *Ranunculus* genus. Genes.

[60] Zhang T (2019). Comparative analysis of the complete chloroplast genome sequences of six species of Pulsatilla Miller, Ranunculaceae. Chin. Med..

[61] Cossard G (2016). Subfamilial and tribal relationships of Ranunculaceae: Evidence from eight molecular markers. Plant Syst. Evol..

[62] He J (2019). Structural variation of the complete chloroplast genome and plastid phylogenomics of the genus Asteropyrum (Ranunculaceae). Sci. Rep..

[63] Korotkova N, Nauheimer L, Ter-Voskanyan H, Allgaier M, Borsch T (2014). Variability among the most rapidly evolving plastid genomic regions is lineage-specific: Implications of pairwise genome comparisons in *Pyrus* (Rosaceae) and other angiosperms for marker choice. PLoS One.

[64] Sun J (2020). Evolutionary and phylogenetic aspects of the chloroplast genome of *Chaenomeles* species. Sci. Rep..

[65] Chi X, Wang J, Gao Q, Zhang F, Chen S (2018). The complete chloroplast genomes of two *Lancea* species with comparative analysis. Molecules.

[66] Parvathy ST, Udayasuriyan V, Bhadana V (2022). Codon usage bias. Mol. Biol. Rep..

[67] Wang R-J (2008). Dynamics and evolution of the inverted repeat-large single copy junctions in the chloroplast genomes of monocots. BMC Evol. Biol..

[68] Maréchal A, Brisson N (2010). Recombination and the maintenance of plant organelle genome stability. New Phytol..

[69] Rao R (2022). The complete chloroplast genome of *Ranunculus*
*yunnanensis* (Ranunculaceae). Mitochondrial DNA Part B.

[70] Raubeson LA (2007). Comparative chloroplast genomics: Analyses including new sequences from the angiosperms *Nuphar*
*advena* and *Ranunculus*
*macranthus*. BMC Genomics.

[71] Marcel D, Sidonie B, Sylwia S, Hanno S, Aurélien T (2017). Mutation rates in seeds and seed-banking influence substitution rates across the angiosperm phylogeny. bioRxiv.

[72] Huang H, Shi C, Liu Y, Mao S-Y, Gao L-Z (2014). Thirteen *Camellia* chloroplast genome sequences determined by high-throughput sequencing: Genome structure and phylogenetic relationships. BMC Evol. Biol..

[73] Daniell H, Lin C-S, Yu M, Chang W-J (2016). Chloroplast genomes: Diversity, evolution, and applications in genetic engineering. Genome Biol..

[74] Henriquez CL (2020). Evolutionary dynamics of chloroplast genomes in subfamily Aroideae (Araceae). Genomics.

[75] Wang W, Messing J (2011). High-throughput sequencing of three Lemnoideae (duckweeds) chloroplast genomes from total DNA. PLoS One.

[76] Abdullah (2020). Complete chloroplast genomes of *Anthurium*
*huixtlense* and *Pothos*
*scandens* (Pothoideae, Araceae): Unique inverted repeat expansion and contraction affect rate of evolution. J. Mol. Evol..

[77] Asaf S (2017). Chloroplast genomes of *Arabidopsis*
*halleri* ssp. *gemmifera* and *Arabidopsis*
*lyrata* ssp. *petraea*: Structures and comparative analysis. Sci. Rep..

[78] Powell W, Morgante M, McDevitt R, Vendramin G, Rafalski J (1995). Polymorphic simple sequence repeat regions in chloroplast genomes: Applications to the population genetics of pines. Proc. Natl. Acad. Sci..

[79] Clark CM, Wentworth TR, O'Malley DM (2000). Genetic discontinuity revealed by chloroplast microsatellites in eastern North American Abies (Pinaceae). Am. J. Bot..

[80] Huang J (2015). Development of chloroplast microsatellite markers and analysis of chloroplast diversity in Chinese jujube (*Ziziphus*
*jujuba* Mill.) and wild jujube (*Ziziphus*
*acidojujuba* Mill.). PLoS One.

[81] Mwanzia VM (2019). The complete chloroplast genomes of two species in threatened monocot genus Caldesia in China. Genetica.

[82] Yi X, Gao L, Wang B, Su Y-J, Wang T (2013). The complete chloroplast genome sequence of *Cephalotaxus*
*oliveri* (Cephalotaxaceae): Evolutionary comparison of *Cephalotaxus* chloroplast DNAs and insights into the loss of inverted repeat copies in gymnosperms. Genome Biol. Evol..

[83] Sato S, Nakamura Y, Kaneko T, Asamizu E, Tabata S (1999). Complete structure of the chloroplast genome of *Arabidopsis*
*thaliana*. DNA Res..

[84] Qian J (2013). The complete chloroplast genome sequence of the medicinal plant *Salvia*
*miltiorrhiza*. PLoS One.

[85] Wang W, Lu A-M, Ren Y, Endress ME, Chen Z-D (2009). Phylogeny and classification of Ranunculales: Evidence from four molecular loci and morphological data. Perspect. Plant Ecol. Evol. Syst..

[86] Kim Y-D, Kim S-H, Kim CH, Jansen RK (2004). Phylogeny of Berberidaceae based on sequences of the chloroplast gene ndhF. Biochem. Syst. Ecol..

[87] Soltis DE (2003). Gunnerales are sister to other core eudicots: Implications for the evolution of pentamery. Am. J. Bot..

[88] Park JM, Oh A, Koo J (2023). Complete chloroplast genome sequence of *Eranthis*
*byunsanensis* BY Sun (Ranunculaceae), an endemic species in Korea. Mitochondrial DNA Part B.

[89] Johansson JT, Jansen R (1993). Chloroplast DNA variation and phylogeny of the Ranunculaceae. Plant Syst. Evol..

[90] Johansson JT (1995). Systematics and Evolution of the Ranunculiflorae.

[91] Cossard G (2016). Subfamilial and tribal relationships of Ranunculaceae: Evidence from eight molecular markers. Plant Syst. Evol..

[92] Hoot SB, Kramer J, Arroyo MT (2008). Phylogenetic position of the South American dioecious genus *Hamadryas* and related Ranunculeae (Ranunculaceae). Int. J. Plant Sci..

[93] Hoot SB (1995). Systematics and Evolution of the Ranunculiflorae.

[94] Wang W, Hu H, Xiang X-G, Yu S-X, Chen Z-D (2010). Phylogenetic placements of Calathodes and Megaleranthis (Ranunculaceae): Evidence from molecular and morphological data. Taxon.

[95] Jin J-J (2018). GetOrganelle: A simple and fast pipeline for de novo assembly of a complete circular chloroplast genome using genome skimming data. BioRxiv.

[96] Shi L (2019). CPGAVAS2, an integrated plastome sequence annotator and analyzer. Nucleic Acids Res..

[97] Schattner P, Brooks AN, Lowe TM (2005). The tRNAscan-SE, snoscan and snoGPS web servers for the detection of tRNAs and snoRNAs. Nucleic Acids Res..

[98] Kearse M (2012). Geneious Basic: An integrated and extendable desktop software platform for the organization and analysis of sequence data. Bioinformatics.

[99] Zheng S, Poczai P, Hyvönen J, Tang J, Amiryousefi A (2020). Chloroplot: An online program for the versatile plotting of organelle genomes. Front. Genet..

[100] Katoh K, Toh H (2010). Parallelization of the MAFFT multiple sequence alignment program. Bioinformatics.

[101] Librado P, Rozas J (2009). DnaSP v5: A software for comprehensive analysis of DNA polymorphism data. Bioinformatics.

[102] Kurtz S (2001). REPuter: The manifold applications of repeat analysis on a genomic scale. Nucleic Acids Res..

[103] Beier S, Thiel T, Münch T, Scholz U, Mascher M (2017). MISA-web: A web server for microsatellite prediction. Bioinformatics.

[104] Benson G (1999). Tandem repeats finder: A program to analyze DNA sequences. Nucleic Acids Res..

[105] Katoh K, Standley DM (2013). MAFFT multiple sequence alignment software version 7: Improvements in performance and usability. Mol. Biol. Evol..

[106] Darriba D, Taboada GL, Doallo R, Posada D (2012). jModelTest 2: More models, new heuristics and parallel computing. Nat. Methods.

[107] Wilgenbusch JC, Swofford D (2003). Inferring evolutionary trees with PAUP. Curr. Protoc. Bioinform..

[108] Rambaut, A. FigTree v1. 3.1. http://tree.bio.ed.ac.uk/software/figtree/ (2009).

