# Supplementary material for: Genetic characterization and phylogenetic analysis of the Nigella sativa (black seed) plastome
Source: Sci Rep. 2024 Jun 24;14:14509. doi: 10.1038/s41598-024-65073-6 (PMC11196742; doi:10.1038/s41598-024-65073-6)
Supplement: Supplementary file 1 — Supplementary Table S1. [file 41598_2024_65073_MOESM1_ESM.docx]

**Table S1.** Analysis of codon bias in the plastome of *N. sativa* species.

| **Amino acid** | **Codon** | **No of codons** | **RSCU** | **Amino acid** | **Codon** | **No of codons** | **RSCU** |
| --- | --- | --- | --- | --- | --- | --- | --- |
| Leucine | UUA | 1068 | 1.3 | Alanine | GCG | 260 | 0.61 |
| Phenylalanine | UUC | 1372 | 0.82 | Tyrosine | UAU | 1365 | 1.36 |
| Leucine | UUG | 999 | 1.22 | Tyrosine | UAC | 649 | 0.64 |
| Leucine | CUU | 1002 | 1.22 | Histidine | CAU | 930 | 1.44 |
| Leucine | CUC | 754 | 0.92 | Histidine | CAC | 366 | 0.56 |
| Leucine | CUA | 679 | 0.83 | Glutamine | CAA | 1001 | 1.39 |
| Leucine | CUG | 431 | 0.52 | Glutamine | CAG | 443 | 0.61 |
| Isoleucine | AUU | 1725 | 1.21 | Asparagine | AAU | 1725 | 1.39 |
| Isoleucine | AUC | 1053 | 0.74 | Asparagine | AAC | 762 | 0.61 |
| Isoleucine | AUA | 1508 | 1.06 | Lysine | AAA | 1912 | 1.35 |
| Metheionine | AUG | 910 | 0.65 | Lysine | AAG | 925 | 0.65 |
| Valine | GUU | 785 | 1.39 | Asparagine | GAU | 1148 | 1.43 |
| Valine | GUC | 422 | 0.75 | Asparagine | GAC | 453 | 0.57 |
| Valine | GUA | 665 | 1.18 | Glutamic Acid | GAA | 1333 | 1.33 |
| Valine | GUG | 391 | 0.69 | Glutamic Acid | GAG | 668 | 0.67 |
| Serine | UCU | 1117 | 1.36 | Cysteine | UGU | 699 | 1.21 |
| Serine | UCC | 933 | 1.13 | Cysteine | UGC | 461 | 0.79 |
| Serine | UCA | 906 | 1.1 | Tryptophan | UGG | 733 | 1 |
| Serine | UCG | 666 | 0.81 | Arginine | CGU | 443 | 0.76 |
| Proline | CCU | 670 | 1.04 | Arginine | CGC | 303 | 0.52 |
| Proline | CCC | 701 | 1.09 | Arginine | CGA | 622 | 1 |
| Proline | CCA | 782 | 1.21 | Arginine | CGG | 421 | 0.73 |
| Proline | CCG | 430 | 0.67 | Serine | AGU | 739 | 0.9 |
| Threonine | ACU | 739 | 1.23 | Serine | AGC | 574 | 0.7 |
| Threonine | ACC | 629 | 1.04 | Arginine | AGA | 1031 | 1.78 |
| Threonine | ACA | 650 | 1.08 | Arginine | AGG | 655 | 1.13 |
| Threonine | ACG | 391 | 0.65 | Glycine | GGU | 521 | 0.91 |
| Alanine | GCU | 569 | 1.32 | Glycine | GGC | 395 | 0.69 |
| Alanine | GCC | 404 | 0.94 | Glycine | GGA | 807 | 1.41 |
| Alanine | GCA | 486 | 1.13 | Glycine | GGG | 568 | 0.99 |
